# Exosomal levels of miRNA-21 from cerebrospinal fluids associated with poor prognosis and tumor recurrence of glioma patients

**DOI:** 10.18632/oncotarget.4699

**Published:** 2015-08-10

**Authors:** Rui Shi, Pei-Yin Wang, Xin-Yi Li, Jian-Xin Chen, Yan Li, Xin-Zhong Zhang, Chen-Guang Zhang, Tao Jiang, Wen-Bin Li, Wei Ding, Shu-Jun Cheng

**Affiliations:** ^1^ Department of Oncology, Beijing Shijitan Hospital, Capital Medical University, Beijing, China; ^2^ Department of Neurosurgery, The First Affiliated Hospital of Xinxiang Medical University, Xinxiang Medical University, Henan, China; ^3^ Beijing Key Laboratory for Tumor Invasion and Metastasis, Cancer Institute of Capital Medical University, Beijing, China; ^4^ University of South Florida, Tampa, FL, USA; ^5^ Department of Neurosurgery, Tiantan Hospital, Capital Medical University, Beijing, China; ^6^ Department of Biochemistry, Molecular Biology Capital Medical University, Beijing, China

**Keywords:** extracellular vesicles, cerebrospinal fluid, hsa-mir-21, glioma, cancer prognosis

## Abstract

Glioma is a most common type of primary brain tumors. Extracellular vesicles, in the form of exosomes, are known to mediate cell–cell communication by transporting cell-derived proteins and nucleic acids, including various microRNAs (miRNAs). Here we examined the cerebrospinal fluid (CSF) from patients with recurrent glioma for the levels of cancer-related miRNAs, and evaluated the values for prognosis by comparing the measures of CSF-, serum-, and exosome-contained miR-21 levels. Samples from seventy glioma patients following surgery were compared with those from brain trauma patients as a non-tumor control group. Exosomal miR-21 levels in the CSF of glioma patients were found significantly higher than in the controls; whereas no difference was detected in serum-derived exosomal miR-21 expression. The CSF-derived exosomal miR-21 levels correlated with tumor spinal/ventricle metastasis and the recurrence with anatomical site preference. From additional 198 glioma tissue samples, we verified that miR-21 levels associated with tumor grade of diagnosis and negatively correlated with the median values of patient overall survival time. We further used a lentiviral inhibitor to suppress miR-21 expression in U251 cells. The results showed that the levels of miR-21 target genes of PTEN, RECK and PDCD4 were up-regulated at protein levels. Therefore, we concluded that the exosomal miR-21 levels could be demonstrated as a promising indicator for glioma diagnosis and prognosis, particularly with values to predict tumor recurrence or metastasis.

## INTRODUCTION

Gliomas are the most common types of human primary tumors of the central nervous system, and are notorious for their aggressive proliferation. Glioblastoma multiforme (GBM) patients typically continue to receive periodical chemotherapy and regional radiation following surgeries, yet the median survival time rarely exceeds two years [1]. This devastating situation has not significantly improved for decades.

The main challenges in glioma patient care and treatments include the precise diagnosis and the timely/comprehensive monitoring of treatment responses. Much of these can be achieved through magnetic resonance imaging (MRI) and routine clinical examinations, however, current forms of assessment have notable limitations. For example, the evaluation of disease progression as manifested during a neurological examination can only give meaningful results when the tumor advanced certain stages with detectable changes. The current resolution limit for MRI remained to be only about 2∼3 millimeters [2, 3], and the subsequent biopsy analyses cannot be repetitively performed and often associate with increased surgical risks.

Exosomes are extracellular membranous vesicles that have been identified and studied for about at least three decades. Recently, much attention of the exosome-related researches has focused to their potential diagnostic and therapeutic values, especially in cancers [4, 5, 6]. Exosomes are small vesicles between a range of 40~100 nm in size, which can be secreted from multiple cell types, such as T-lymphocytes, neurons, and malignant brain tumor cells. Exosomes exist and circulate in the body fluids, containing and transporting a broad spectrum of nucleotide and protein species, and can be used as a mechanism for intercellular communication of both healthy and cancerous cells [7, 8].

MicroRNAs (miRNAs) are a class of small non-coding RNA molecules that regulate the expression of specific target gene by modulating the the processes of both gene transcription and protein translation. Numerous studies have shown that miRNAs were able to regulate the cellular levels of oncogenesis/tumor suppressors and their mediated signal transduction pathways. Recently, several miRNAs detected in the patients’ serum were suggested to predict the presence of malignant astrocytomas. Meanwhile, tumor-derived exosomes have been demonstrated with even greater potential to predict tumor progression, relapse, and treatment failure. Using quantitative real-time polymerase chain reaction (qRT-PCR) methods, earlier investigations demonstrated that the miR-21 expression in glioma tissue samples was significantly higher as compared to normal or paracancerous tissues [8]. The levels of miR-21 were also correlated with the histological grade.

The exosome RNAs contained cancer-derived tissue specific miRNAs in relative high abundance. These tissue-specific miRNAs were known to associate with the development of specific types of tumors. The measurement of exosomal miRNA levels could be useful for providing important information for the diagnosis and prognosis of specific cancer types, including gliomas [8, 9, 10]. Hence, to systematically evaluate whether exosome-derived tumor-specific miRNAs of representative types can be used directly from clinical body fluid samples and exert values for the evaluation of patient treatments is indeed an immediate and interesting question to be addressed

MiR-21 has drawn great attentions due to its diagnostic as well as prognostic values in a various types of cancer, including glioma. The level of miR-21 was up-regulated in the tissue of higher grade gliomas (grade III and glioblastomas) compared with that of the non-neoplastic brain tissues and promote cell survival and invasion in glioblastoma cells [11, 12] A range of target genes have been identified for miR-21, among which, PTEN [13], RECK4 [12], PDCD4 [14] have been shown to closely related to the functionality of miR-21 in growth and metastasis of gliomas. However, whether the regulatory machinery of miR-21 on glioma still functions in the form of CSF exosomes remains elusive.

In this study, we hypothesized that exosomal miR-21 from the CSF of glioma patients may be a reliable, robust and practical index for the assessment of tumor progression and prognosis. Based on a collection of serum and CSF samples from in-ward post-surgery patients, we first established a method to prepare high-grade exosomes as determined by transmission electron microscopy (TEM) and western blotting. We then determined the miR-21 expression for the correlation with clinical parameters in comparison to the serum or non-exosomal total levels. The role of miR-21 in glioma cells was also investigated using a lenti-virus-mediated knockdown system in *in vitro* experiments. We were able to demonstrate that the exosomal miR-21 levels in CSF of glioma patients associated with tumor recurrence.

## RESULT

### Morphological and biochemical characterization of the extracellular vesicles prepared from the body fluids of glioma patients

**Figure 1:**
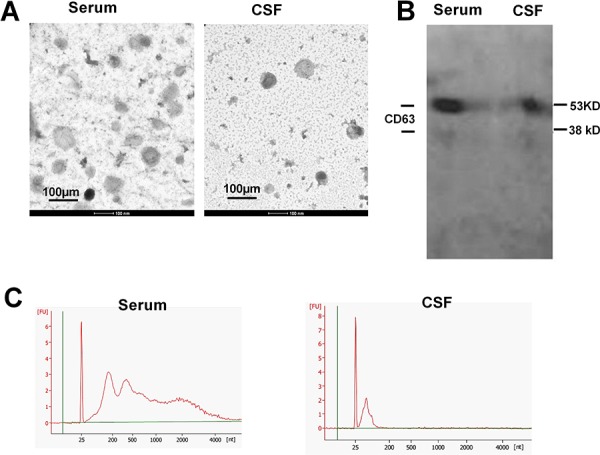
Characterization of the extracellular vesicles **A.** Transmission electronic microscopy of the preparation from patients’ serum and cerebrospinal fluids. Scale bars, 100 nm. **B.** Western blotting detection for exosomal marker CD63. **C.** The spectrum from an Agilent Bioanalyzer using RNA extracted from both serum exosomes and CSF exosomes

At the present time, several methods to isolated exosomes are available with differences in yields and qualities. In this study, we adopted a traditional ultracentrifugation protocol as a standard operation procedure (SOP) for preparing exosomes from body fluids. The obtained extracellular vesicles (EVs) were suspended and subjected to TEM examination to identify the qualities. The EVs from serum and CSF (Figure 1A) resembled in morphological characters as spherical structures with diameters around 100 nm. To verify whether these vesicles contained exosomal markers, we used Western blotting to probe CD63 molecules (Figure 1B). Two specific bands were detected at 53 and 38 kD. As the exosomes were known to carry various substances, such as receptors, phosphatidyl choline, mRNA and miRNA, etc., we examined the ribonucleic acid contents in our prepared exosomes using a high sensitive bioanalyzer (Figure 1C). The results showed that the exosomes from CFS exerted a different profile from those derived from the serum, with much less total RNA abundance, however with much higher enrichment in the molecular range of miRNAs. The isolated vesicles fitted the consensus profile of exosomes [15] with sufficient qualities and were suitable for subsequent laboratory assays.

### Determination of exosomal miR-21 expression in glioma and non-glioma patients

The expression of miR-21 was known to associate with the progression of many types of tumors, including gliomas. Nonetheless, the exosomal miR-21 was less characterized. [16, 17, 18, 19] We have collected the serum and CSF samples from 70 patients, and compared the miR-21 levels with a set of non-tumor controls.

**Figure 2:**
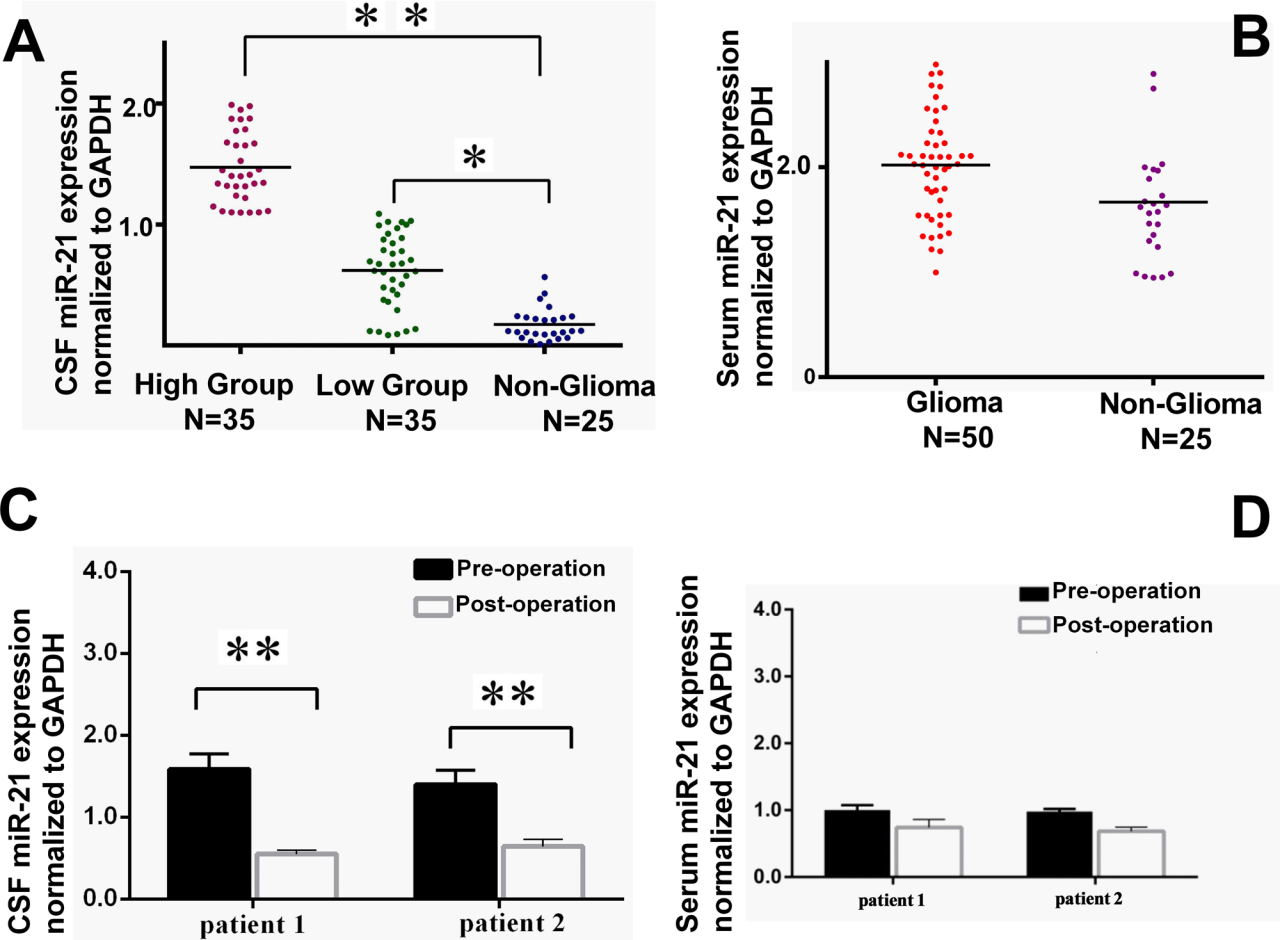
Comparison of the exosomal miR-21 levels in CSF (*n* = 70) or serum (*n* = 50) samples from low- and high-grade glioma patients with the non-glioma controls Meanwhile, **A.** Relative CSF miR-21 levels to GAPDH in low-grade (*n* = 35) and high-grade glioma groups (*n* = 35) versus in non-glioma patients (*n* = 25). **B.** Serum exosomal miR-21 in glioma patients (*n* = 50) versus non-glioma patients (*n* = 25). **C.** Comparison of CSF exosomal miR-21 level and **D.** Serum exosomal miR-21 between pre-surgery and post-surgery in two patients underwent the second operation due to tumor recurrence. *p < 0.05 and **p < 0.01.

The patients included in this study were described as in Table 1. Additional information was provided (Supplementary Table S1). qRT-PCR was used to measure miR-21 abundance [20], and the results were normalized to GAPDH as the reference. The expression of miR-21 from the CSF exosomes were significantly elevated in both high and low-grade glioma groups than that of the control with *p* < 0.01 and 0.05, respectively (Figure 2A). No significant difference of the miR-21 level from serum exosomes was found between glioma and non-glioma groups (Figure 2B). This indicated that exosomes appeared to be better sources for miR-21 detection to differentiate glioma patients for non-tumor diseases.

**Table 1:** Clinical features of glioma patients with high and low exosomal miR-21 levels as separated by the median value

	Exosomal miR-21: Number of patients			
	Total number	High Expression Group	Low Expression Group	*P*
**Total number**	70	35	35	
**Sex (female/male)**	70	14/21	11/24	***1.000***
**Age at diagnosis (>35 year)**		27	32	***1.000***
**Histology**				
I/II	25	12	10	
III/IV	45	23	25	***0.609***
**Extent of resection (Gross/Sub-total/biopsy)**	70	17/10/8	19/8/8	***0.729***
MGMT (L/H)	50	13/11	11/15	***0.406***
Ki-67 (L/H)	42	13/8	14/7	***0.750***
PTEN (L/H)	50	15/10	12/13	***0.399***
MMP-9 (L/H)	45	19/6	13/7	***0.424***
EGFR (L/H)	45	10/9	14/11	***0.826***
Mutant P53 (L/H)	54	16/8	20/10	***1.000***
*MGMT* promoter methylation (U/M)	25	4/10	4/7	***0.685***
**Tumor side (L/B/R)**	70	14/13/8	20/9/6	***0.193***
**Site of the primary tumor (Include this lobe/not)**				
Frontal lobe	25	12/20	13/20	***0.876***
Temporal lobe	31	16/21	15/24	***0.674***
Parietal lobe	12	8/17	4/18	***0.284***
Occipital lobe	5	4/19	1/21	***0.175***
Ventricle	11	7/21	4/21	***0.424***
Spinal	10	7/20	3/20	***0.261***
Others	14	6/20	8/21	***0.704***

Abbreviations: *U*, unmethylated; *M*, methylated; protein expression: *L*, low; *H*, high; side: *L*, left; *B*, both; *R*, right; SD, standard deviation; *y*, year; *EGFR*, epidermal growth factor receptor; *MGMT* O6-methylguanine-DNA methyltransferase; *P < 0.05.

To evaluate other factors that might affect the outcomes of the glioma patients involved in this study, clinical records for known neuro-pathological molecules [21] were analyzed. The prognostic markers, including (Ki-67; *n* = 42), epidermal growth factor receptor (EGFR; *n* = 45), O6-methylguanine-DNA methyltransferase promoter methylation (MGMT; *n* = 25), phosphatase and tensin homolog (PTEN; *n* = 50), matrix metalloproteinase (MMP; *n* = 45), and P53 mutations (P53; *n* = 54), were summarized in Table 2. The basic clinical information such as age, sex, histology, extent of resection, primary tumor location, and surgical operation notes were used as referring variables subjected for statistical analyses. No difference was found between in the high and low miR-21 two groups.

**Table 2:** Prognostic assessment of exosomal miR-21 expression with 3clinical features for recurrent glioma patients

		Exosomal miR-21: No. of Patients		
	Total No	High Expression Group	Low Expression Group	*P*
**Pattern Of Recurrence**				
Local Tumor bed	37	10	27	
Diffuse intracranial or/and spinal dissemination	33	25	8	**<0.001**
**Interval To Recurrence (Median ± SD, m)**		26.4 ± 22.0	23.0 ± 20.7	*0.432*
**Treatment**				
Re-operation	6	1	5	
Chemotherapy	64	34	30	*0.09*
**Overall follow-up time(months)**		2–90	1–102	
**Survival**				
Alive	42	12	25	**0.002**
Deceased	28	23	10	

Abbreviations: SD, standard deviation; m, month; *P* < 0.05.

In this study, two special patients have received special attentions, both of which were receiving a second surgery in our hospital. Their primary glioblastoma were removed about two years earlier, unfortunately, recent MRIs indicated that the tumors have re-grown at the local site. The CSF and serum pre-operation and post-operation were collected and compared for the exosomal miR-21 expression. The results showed the exosomal miR-21 drastically decreased after the surgical operations. But no difference was detected in the serum samples pre- or post-surgeries (Figure 2C and 2D). It inferred that the source of elevated miR-21 was directly from the tumor, and the CSF measures for miR-21 could be better representing the tumor-derived miR-21 levels.

### ROC and Kaplan-Meier analyses for the diagnosis and prognosis values of tumor derived miR-21 levels in glioma patients

**Figure 3:**
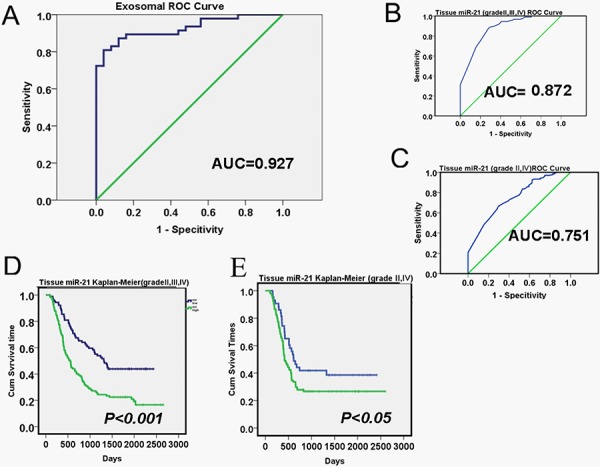
Diagnostic and prognostic values of exosomal miR-21 levels in glioma patients **A.** Receiver operation Characterization (ROC) curves based on exosomal miR-21 levels to differentiate recurrent glioma patients (*n* = 70) from non-tumor diseases. The area under curve (AUC) was as 0.927 (95% CI: 0.865–0.985). The ROC curves to discriminate low grade (Grade II) glioma and high grade (Grade IV or Grade III) cases were shown in **B** and **C.** with the AUC of 0.872 (95% CI: 0.817–0.927) and 0.751 (95% CI: 0.681–0.821), respectively. **D** and **E.** Kaplan-Meier survival statistics using microarray detection of tissue miR-21 abundance for glioma prognosis (*n* = 198, from CGGA).

To evaluate the diagnostic values of exosomal miR-21 for tissue glomas, we detailed the basic characteristics of these patients (Supplementary Table S2) Receiver Operatiog Characteristics (ROC) curves were generated to discriminate glioma/non-glioma patients or gliomas in different grades. The area under curve (AUC) for exosomal miR-21 was 0.927 (95% CI: 0.865–0.985), which appeared to be an excellent index to differentiate glioma and non-tumor brain diseases (Figure 3A). When using documented microarray measures of the tissue samples from CGGA (Relative level of miR-21 were shown in Supplementary Figure S2), the AUC from 198 patients was 0.872 (95% CI: 0.817–0.927) for the discrimination of grade III/VI from grade II gliomas. To specifically separate grade II and grade VI gliomas, the AUC was 0.751 (95% CI: 0.681–0.821) (Figure 3B and 3C). Thus, the miR-21, especially the exosomal miR-21 was from the CSF was seemingly exerted better diagnostic values, even in comparison with the measures from the primary tumor tissues. Besides for diagnostics, according to CGGA matched follow-up records, from 198 patients with 15% current survival, the Kaplan-Meier analysis also indicated that tissue miR-21 levels also have prognositic values (Figure 3D and 3E). The results to separate glioma patients of benign (grade II) and malignant (grade VI) gliomas were indicated with significant differences.

To identify any correlations between miR-21 expression and glioma recurrence, we compared the anatomical site of recurrence, time to recurrence, and relapse interference. The percentage of patients with diffuse intracranial, diffuse spinal dissemination, diffuse intracranial, and spinal dissemination was significantly difference between the high miR-21 and low groups with *p* < 0.001 (Table 2). Additional Cox analyses were conducted for prognostics with factors of World Health Organization (WHO) grade, histology, gender, age, and Karnofsky performance status (KPS) using records extracted from the CGGA database (Supplementary Table S3).

### Suppression of miR-21 expression in glioma cells altered both cellular and exosomal expression of miR-21 targeted cancer-associated genes and induced cell apoptosis

**Figure 4:**
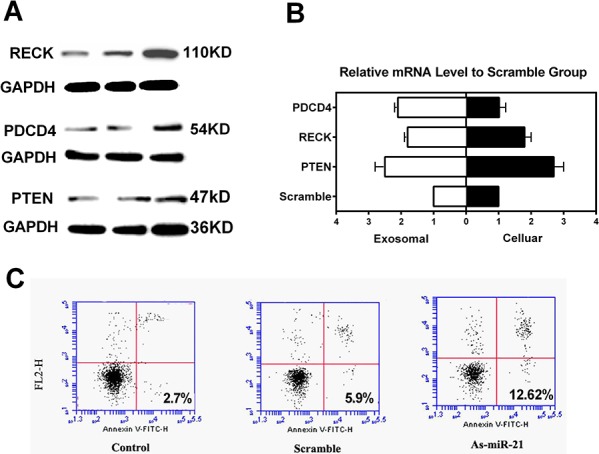
Effects of miR-21 depletion in U251 glioma cells **A.** Western blotting of miR-21 target genes of RECK, PDCD4 and PTEN following miR-21 inhibition at 48 h. **B.** Cellular and exosomal mRNA levels of PTEN, RECK and PDCD4 as determined by RT-qPCR (*p < 0.05, **P < 0.01). **C.** Flow cytometry of Annexin V staining for the evaluation of cell apoptosis following miR-21 inhibition.

To explore the underlining mechanisms of miR-21 in glioma progression and rationalize the potential applications on miR-21 to be used as a clinical diagnostic reference, we used an experimental approach of miR-21 inhibition for *in vitro* analyses of selected miR-21 targeted genes. By applying lentivirual based vectors in U251 glioma cells lines, we probed anti-oncogenic proteins of PDCD4, RECK and PTEN at both protein and mRNA levels for their expression. As compared to a scramble control, we found that the expression of these genes was significantly increased upon the suppression of miR-21 levels (Figure 4A and 4B). We also collected cell culture medium following the gene transfer treatments and we were able to demonstrate the consistent increase in the exosomal mRNA expression of the selected miR-21 targeting genes in response to miR-21 inhibition (Supplementary Figure S3). We further assessed the cell apoptosis using Annexin V/PI staining in U251 cells following transfection of miR-21 antogonist and compared with the controls. As shown in Figure 4C, the results showed that miR-21 knockdown led to a substential increase of apoptosis in the treated cell.

## DISCUSSION

The expression of miR-21 has been reported with a high-level profile in the tissue of higher grade gliomas, mostly gliomas of grade III and glioblastomas. As compared with the non-neoplastic brain tissues, the up-regulated miR-21 was suggested to manifest an anti-apoptotic effect in glioblastoma cells [11] Recently, tissue sources of cerebrospinal fluids or plasma from glioma patients began to draw attentions from clinical researchers, for the findings that high levels of miR-21 in these samples often correlated to aggressive phenotypes. [24, 25, 26] Cumulating evidence have suggested the importance of miR-21 levels in body fluids as an indicator of poor prognosis of various cancer types, including gliomas.

Currently, the study of the nucleic acids in extracellular vesicles, predominantly exosomes, has rapidly advanced. The results suggested that levels of miR-21 in the EVs from CSF could be a more promising source to be used for the search of biomarkers to correlate the development of glioblastoma [27] However, the majority of these reports involved only primary glioma patients with limited patient numbers, the diagnostic and the prognostic value of miR-21, especially those using CSF exosomes for microRNA detections, required further clinical validations. Besides, whether CSF-derived or exosomal miRNAs of specific species could be used to evaluate recurrent glioma patients remained unknown and deserve in-depth investigations.

In the present study, we first established a reliable standard protocol to isolate exosomes from the CSF of 70 glioma patients with recurrence. We used CSF exosomal miR-21 as a primary indicator to estimate its value of in recurrent glioma, as compared to the miR-21 levels in the serum. Consistent with the earlier reports in primary glioma patients, the level of exosomal miR-21 was indeed significantly up-regulated compared with other types of non-oncogenic neuropathic patients. The elevated miR-21 levels also seemed to correlate with the clinical grade of glioma malignancy (Fig. 2A).

Various tumors were indicated with high expression of miR-21. The high level of miR-21 in CSF was believed to be originated from the tumor cells. From the comparison between pre- and post-surgery samples or the patients with/without a second surgery, our results supported the conclusion that the tumor-derived RNAs, especially miR-21, were able to represent the status of glioma progression. As demonstrated in Figure 2, CSF could be regarded as a more suitable tissue source for diagnosis, as the serum or plasma contained more non-tumor originated miRNA species. In some reports, it was suggested that exosomal miRNAs were more likely to be tumor-derived, therefore might be used for prognosis. [26] The reverse and attenuated incline of the CSF exosomal, but not serum mir-21 level from the two recurrent patients with second operations strongly suggested that the source exosomal mir-21 was rather from the tumor itself, instead of from other cell types. To this matter, we moved on and compared the total serum miR-21 and serum exosomal miR-21 with the CSF measures. Except in certain specific cases, such as the re-surgeries, serum miR-21 in total nor in exosomes failed to pass the significance threshold to to discriminate glioma and non-glioma patients (Fig. 2B), neither glioma patients with different grades. This was different from some previous report when the total mir-21 levels in plasma from primary glioma patients were examined. We postulated that the variation of miRNA levels in the serum could be very much influenced by patient conditions and the treatment responses. Hence, CSF should be better used for miR-21 examinations. Moreover, the exosomal levels would be preferred for more accurate measurements, in addition to the convenience for normalization and sample preservations.

Directly using the mir-21 level of <0.25 copy/EV from CSF as a discriminating threshold for the classification of glioblastoma has been attempted in primary glioma patients [27] We performed ROC analyses to assess the exosomal mir-21 for an indicator for the diagnosis of recurrent glioma patients. The AUC of 0.927 demonstrated its striking promises in the context of both sensitivity and specificity. This appeared to be significantly improved from the referencing index when CGGA glioma sample records were used for validations, where the AUC of tissue mir-21 for discriminating II/III/IV grade or III/IV grade was 0.751 to 0.872 from 198 patients. As miR-21 were also suggested as a factor for cancer prognosis, we extracted the tissue miR-21 results from CGGA and performed Kaplan-Meier analyses (Figure 3C and 3D). We found that high abundance of miR-21 from glioma tissues defined a worse prognosis in primary glioma patients, and this was in support of previous reports in glioma and other tumors. [27] To this point, it has unarguably raised the importance for detecting the CSF exosomal miR-21 in clinical follow-up studies, as it might provide valuable and critical information, not only for patients’ survival, as well as for the responses during treatments.

Besides monitoring the CSF exosomal miR-21 levels in glioma patients, a list of well-known prognostic glioma-related markers, including levels of Ki-67, EGFR, PTEN, mutant P53, and methylation of the MGMT promoter were worthwhile to be analyses for correlation studies. As shown in Table 2 of the current 70 patients, some of these factor might not predict the reoccurrence by itself, however, it could provide important reference according to certain miR-21 threshold for separated groups of primary glioma patients. Importantly, some of the candidate makers were demonstrated as miR-21 regulated targets.

Using a U251 glioblastoma cell line as an *in vitro* model, we tried to address several questions with the inhibition of miR-21 as specific treatments. These included whether miR-21 levels indeed influence and negatively correlate the levels of its certain targeted genes, whether the exosomal expression of the miR-21 targeted genes correlated with the cellular messenger levels, and the miR-21 changes in either cellular or exosomal levels are sufficient to alter cell apoptosis of glioma cells. Besides miR-21, its target genes of PTEN, PDCD4, RECK were selected, including PTEN as one of the most important tumor suppressors. As expected, both the medium exsomal and intracellular levels of mir-21 targets were up-regulated when miR-21 antagonist was applied in U251 cells. Increased cell apoptosis was detected in the glioblastoma cells under the accorded conditions. Together with the results concerning CSF exosomal mir-21 in recurrent glioma patients, it is quite plausible that increased mir-21 in CSF exosomes could be transported to receiver cells, favoring an oncogenic effect which might contribute to glioma reoccurrence.

Two important findings from the present study, which were different from previous reports, concerned the exact functionality of miR-21 in glioma patients. At first, this appeared to be a very initial report focusing on both the diagnostic and prognostic value of miR-21 on recurrent glioma patients in relative large numbers. Secondly, it demonstrated from multiple aspects for the importance and potential of using CSF exosomal miRNAs as a novel promising approach for the follow-up clinical studies involving glioma patients for the assessment of recurrence. The continous estimation about the diagnostic value of miR-21, especially in the CSF vesicles, might encompass a complex of microvesicles, exosomes, retrovirus-like particles (RLPs) as well as apoptotic bodies. [24] The standard protocol for exosome isolation and identification we developed for this study could provide an example for miRNA detection in CSF exosomes from recurrent glioma patients.

## MATERIALS AND METHODS

### Patients and samples

A total of 70 patients (42 males, 28 females) were included in this study. Patients were diagnosed with glioma, which included eight cases of astrocytoma (WHO II), 25 cases ependymoma (WHO II) and 45 cases of glioblastoma (WHO IV). All had recurrence between 2013/01/01 and 2015/5/31. Besides the clinical symptoms, diagnoses were confirmed by contrast-enhanced MRI and the consensus on histology from at least two neuropathologists at the Department of Oncology in Beijing Shijitan Hospital. The collection of the CSF and blood samples strictly followed ethics protocols and each participant signed consent forms. All patients consented to be registered in the Chinese Glioma Genome Atlas (CGGA) database. Surgical tissue was collected and stored prior to chemotherapy and/or radiation therapies. This study was authorized by the ethics committee of Shijitan Hospital. The use of patients’ medical records on an anonymous basis was granted for this report.

### Sample processing

Blood samples of 5 mL were collected from each patient into serum separator tubes and processed within 60 min. Serum was centrifuged at 3000g for 30 min to remove cells and stored at −80°C. Collection of CSF from glioma and non-tumor brain trauma patients was performed via a lumbar puncture during the surgery. Collected CSF was immediately centrifuged at 500 g for 30 min to remove cells and cell fragments. Supernatants were stored in aliquots at −80°C. The medium were also deal with upper procedures and were stored in −80°C.

### Cell culture and extracellular vesicle (EV) isolation

U251 cells were maintained in DMEM/F12 supplemented with 10% EV-free FBS at 37°C with 5% CO_2_. Transduction was performed when cells reached 50~70% confluence with scramble or miRZip-21 (1 × 10^8^ TU/ml lentivirus) [22, 23] following the manufacturer’s recommendation After 48 hours, the supernatant were collected into 50mL sterile vessels.

The fractionation and purification of exosomes from serum (500 μL), CSF (5–7 mL) or cell culture medium (20–30 mL) were conducted by accorded centrifugation protocols at 4°C. Briefly, the fluid samples were subjected for an initial centrifugation at 2,000 g for 30 min to remove any cellular debris. The cell-free supernatant was then diluted 1:1 with sterile 1× PBS and centrifuged at 12,000 g for 25 min. Samples were then ultracentrifuged at 150,000 g for 90 min. The pellet was resuspended in 200 μL PBS, and stored at −80°C. Further analyses were performed with te avoidance of unnecessary freeze-thaw cycles to avoid influencing the stability of vesicular structures. The obtained samples were analyzed by TEM and biochemical assays for quality controls.

### Transmission electron microscopy (TEM)

Frozen CSF (5 mL), serum (500 μL) or medium (20 mL) were thawed at room temperature and ultracentrifuged at 150, 000 g for 90 min. Pellets containing exosomes were fixed with 100 μL glutaraldehyde. The suspension (~2.5 μL) was mounted onto carbon-coated cooper grids by floating the grid on the droplet for 10 min. Excess liquid was removed with clean, dry filter paper. The grid was then transferred and stained with 0.75% uranyl formate for 30 sec and then dried with filter paper. The grid was placed on a piece of paper for several minutes to dry [21] Samples were imaged on a JEOL1010 TEM. Size measured with image (NIH).

### Western blot analysis

Isolated exosomes were lysed in RIPA buffer (25 mM Tris-HCl pH 7.6; 150 mM NaCl; 1% sodium deoxycholate; 0.1% SDS; 1% NP-40) and centrifuged at 200,000 g for 10 min. Supernatants were collected and heated at 100°C for 5 min, then loaded onto 12% SDS-PAGE gels (Criterion TGX Precast Gel, Bio-Rad Laboratories). Protein was transferred to PVDF filters, blocked with 5% non-fat milk, and incubated overnight with 1:2000 primary monoclonal antibody against CD63 (Abcam), RECK [23] (polyclonal, 1:1000, Abcam), PTEN [22] or PDCD4 [22] (monoclonal, 1:2000, Abcam). The filters were washed three times with PBST and then incubated with HRP-conjugated secondary antibody (Jackson 1:10000). The blots were imaged using a G:BOX instrument (Syngene, USA) for chemiluminescence detection.

### RNA extraction and qRT-PCR quantification of mRNA and microRNA

Total RNA derived from CSF, serum and medium were isolated from the prepared exosomes using an RNeasy serum/plasma Kit (Qiagen, USA), as recommended by the manufacture’s protocol. The quality and yield of each RNA sample was measured using a 2100 Bioanalyzer (Agilent Technologies, Sata Clara, CA) and NanoDrop (NanoDrop Technologies, Houston, TX). RT-qPCR was used to detect both cellular and exosomal mRNAs/miRNA. The primers for GAPDH, miR-21, PTEN, RECK and PDCD4 were purchased from Ambion. RT-PCR parameters were set following the manufacturers’ instructions. The exosomal miR-21 data were normalized to GAPDH and referred as the ratios to the scramble control when being used.

### Prepare extracellular vesicle (EV)-depleted medium

The EV-depleted fetal bovine serum used for this study was prepared by spin fresh fetal bovine serum (FBS) at 120,000 × g for 9 hours at 4°C. The supernatant was then collected into a disinfectant vessels and reacted with 25 ml ExoQuickTC (System Biosciences, USA) per each of 100 mL for incubation overnight at 4°C. Following a final centrifugation procedure at 1500 × g for 30 minutes at 4°C, the resulting supernatant was collected and ready to supplement DMEM/F12 at 10% as EV-free medium for the culture of U251 cells.

### Lentiviral based inhibition of miR-21 in U251 cells

U251 glioblastoma cells cultured in 6-well plates were transfected with a pmiRZip-21 plasmid (Genepharm, Shanghai, China) or infected with the produced lentivirual vector for suppressing the level of miR-21 [23] expression. The expression of the miR-21 targeted genes of PTEN, PDCD4 and RECK at 48h were detected at both transcriptional and translational level by qRT-PCR and Western blotting. Flow cytometric analyses were employed to assess cell apoptosis at 24h following the treatments for miR-21 inhibition.

### Flow cytometry analyses of apoptosis

U251 cells were collected and rinsed by consecutive centrifugation of 500g × 10 min at 4°C for 2 rounds. The cells were then resuspended in a staining buffer with the then addition of 10 μL Annexin V-FITC and 5 μL propidium iodide (PI). After incubation at room temperature for 13 min, the cells were subjected to flow cytometric analyses using a (FA CSC, BectonDickinson USA) system.

### Statistical analysis

Presented values are the averages of triplicate samples. To compare miR-21 expression levels, two-sided *x*^2^ tests were used. Statistical significance was set at *P* < 0.05. Student *t* tests were performed to analyze the difference in clinical factors such as patients’ ages. Survival analysis was assessed by the Kaplan-Meier method. The COX regression analysis was employed to estimate the risk factor of gliomas. We used receiver operating characteristic (ROC) curves to assess the specificity and sensitivity between the exosomal miR-21 and microarray data of tissue sample. The SPSS statistical software package (version 13.0) was used for single- or multi-factorial analyses and the charts were created using GraphPad Prism V6 for Windows.

## SUPPLEMENTARY FIGURES AND TABLES


