# Supplementary material for: Exosomal levels of miRNA-21 from cerebrospinal fluids associated with poor prognosis and tumor recurrence of glioma patients
Source: Oncotarget. 2015 Aug 10;6(29):26971–81. doi: 10.18632/oncotarget.4699 (PMC4694967; doi:10.18632/oncotarget.4699)
Supplement: Supplementary file 1 [file oncotarget-06-26971-s001.pdf]

## SUPPLEMENTARY FIGURES AND TABLES

A

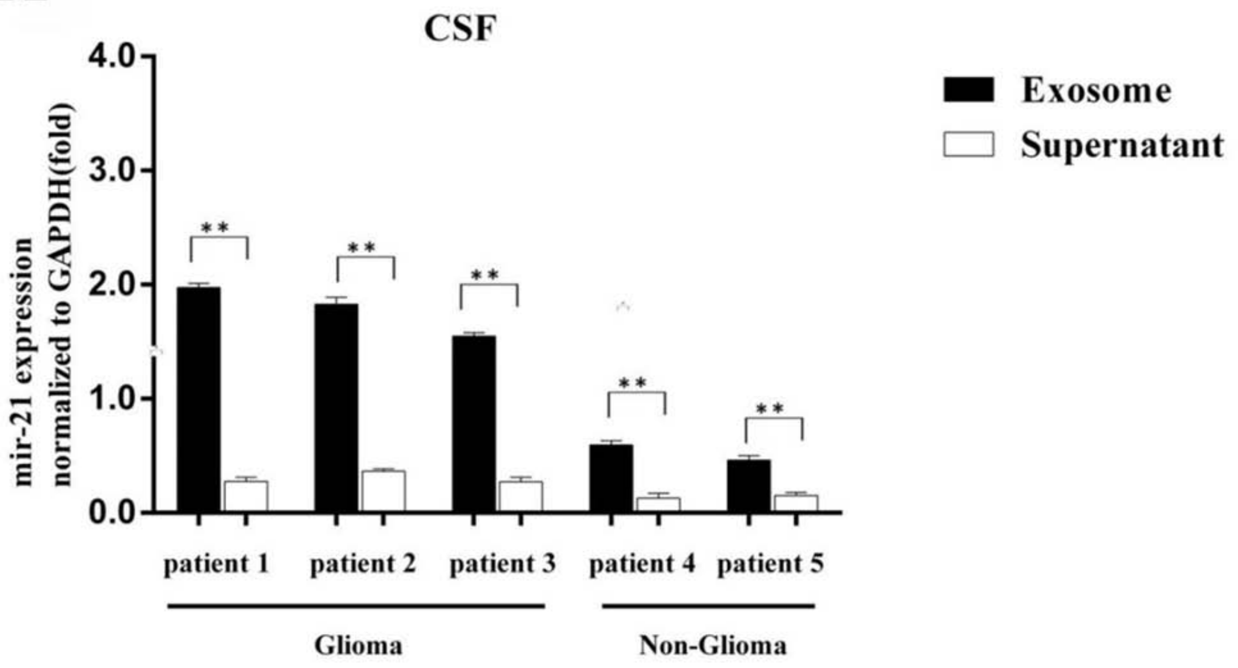


B

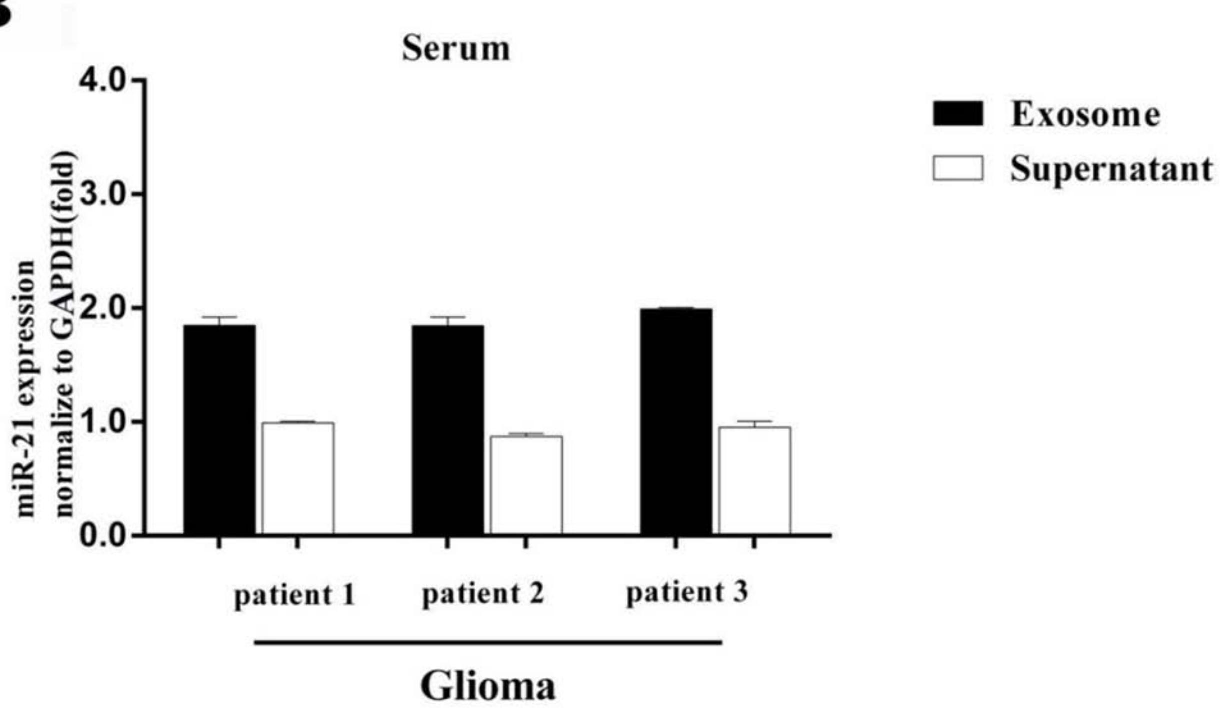


**Supplementary Figure S1: miR-21 expression levels. A.** miR-21 levels in CSF of glioma or non-glioma control patients. **B.** miR-21 levels in serum of the glioma patients. ***p* < 0.01.

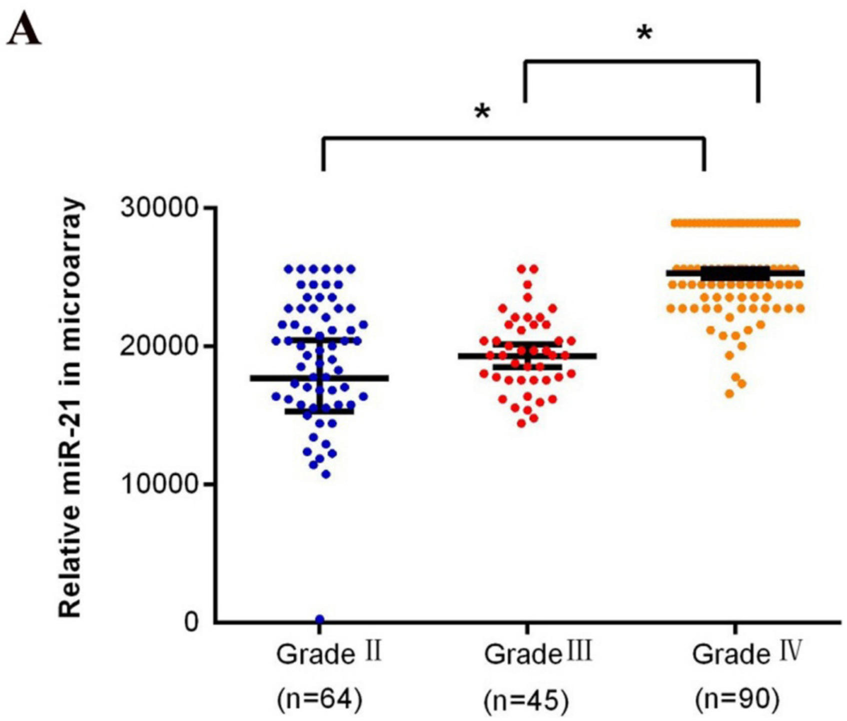


**Supplementary Figure S2: Microarray analysis of miR-21 in glioma patients of different glioma grades.** Relative miR-21 expression in brain tissues of 198 glioma patients with pathological grades of II–IV.

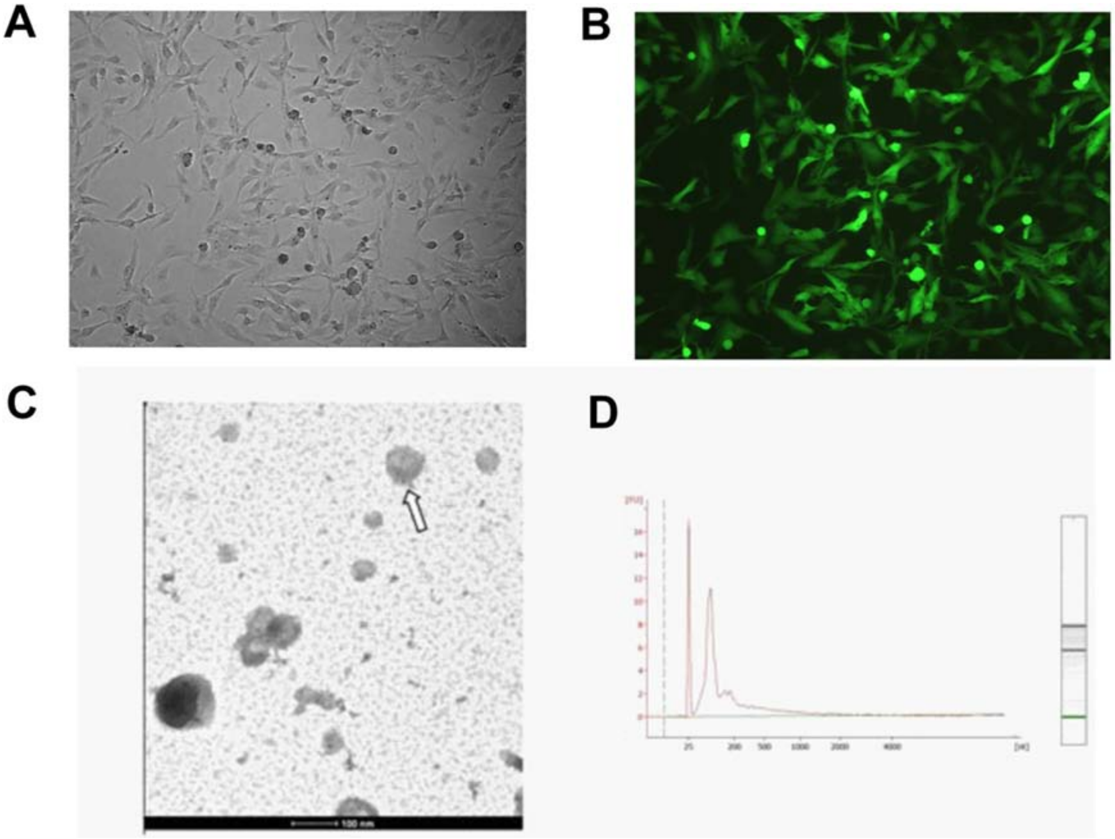


**Supplementary Figure S3: Lentiviral infection of GFP construct into U251 cells. A.** U251 cells under bright field of the microscope. **B.** U251 cells infected by Lentiviral–GFP with Roche Fugene HD for 48 h. **C.** TEM images of exosomes derived from the supernatant of U251 medium for identification of exosomes. **D.** Quality analysis of exosomal RNA of the cell determined by an Agilent Bioanalyzer.

**Supplementary Table S1. Clinical features of patients with glioma or non-glioma controls**

| | % of patients | | |
|---|---|---|---|
| | **Control Group** | **Glioma Group** | ***P*** |
| Total number | 25 | 70 | |
| Men | 18 (72) | 42 (60) | |
| Women | 7 (28) | 28 (40) | ***0.944*** |
| Age: Median±SD | 54.3 ± 16.9 | 49.8 ± 13.5 | ***0.452*** |
| Reasons for admission | | | |
| Cerebral hemorrhage | 3 (12) | | |
| Hydrocephalus | 1 (4) | | |
| Severe head trauma | 10 (40) | | |
| Subarachnoid hemorrhage | 7 (28) | | |
| Pituitary adenomas | 2 (8) | | |
| Lymphocyte infiltration hypophysitis | 2 (8) | | |

Abbreviations: SD, standard deviation; $P < .05$.

**Supplementary Table S2. Correlation of miR-21 expression level with other clinical pathological features in 198 glioma patients**

| Clinicopathological features | No. of cases | miR-21 expression | | *P* |
|---|---|---|---|---|
| | | Low (*n*, %) | High (*n*, %) | |
| **WHO grade** | | | | <0.001 |
| **II** | 64 | 47 (73%) | 17 (17%) | |
| **III** | 45 | 38 (84%) | 7 (16%) | |
| **IV** | 90 | 13 (14%) | 77 (86%) | |
| **Sex** | | | | 0.23 |
| **Male** | 133 | 58 (43%) | 75 (57%) | |
| **Female** | 65 | 40 (61%) | 25 (39%) | |
| **Age** | | | | <0.001 |
| **<50** | 48 | 23 (47%) | 27 (53%) | |
| **>50** | 150 | 25 (16%) | 125 (84%) | |
| **KPS** | | | | 0.373 |
| **>70** | 70 | 38 (54%) | 32 (46%) | |
| **<70** | 128 | 60 (46%) | 68 (54%) | |

## Supplementary Table S3: Cox multivariate analysis

| Parameter | Risk | 95% confidence interval | *P* |
|---|---|---|---|
| Age | 1.200 | 0.634–1.212 | 1.232 |
| Sex | 1.227 | 0.810–1.857 | 1.227 |
| KPS | 0.850 | 0.572–1.264 | 0.850 |
| WHO grade | 3.543 | 2.637–4.760 | 0.000 |
| miR-21 xpression level | 1.634 | 1.083–2.467 | 0.019 |
